# Patient-Reported Outcomes Measurement Information System Validation in Hip Arthroscopy: A Shift Towards Reducing Survey Burden

**DOI:** 10.7759/cureus.13265

**Published:** 2021-02-10

**Authors:** Erik Gerlach, Ryan Selley, Daniel Johnson, Richard Nicolay, Gregory Versteeg, Mark Plantz, Vehniah Tjong, Michael Terry

**Affiliations:** 1 Department of Orthopaedics, Northwestern University Feinberg School of Medicine, Chicago, USA

**Keywords:** hip arthroscopy, femoral acetabular impingement, promis, computer adaptive testing, validation

## Abstract

Background

The Patient-Reported Outcomes Measurement Information System (PROMIS) was developed to provide measures of patient-reported symptoms and healthcare outcomes across a variety of conditions in an easily accessible manner. The purpose of this study was to validate PROMIS against traditional legacy measures in patients undergoing hip arthroscopy for femoral acetabular impingement (FAI).

Methodology

Outcome measures collected pre- and post-operatively included PROMIS Pain Interference (PI) and Physical Function (PF), modified Harris Hip Score (mHHS), Hip Outcome Score-Activities of Daily Living and Sport (HOS-ADL and HOS-Sport), Nonarthritic Hip Score (NAHS), and Visual Analog Scale (VAS). Pearson’s correlation coefficients were calculated between each outcome measure.

Results

Strong correlations were observed between the PROMIS PF T-Score and the mHHS (r = 0.64-0.83, p < 0.0001), HOS-ADL (r = 0.54-0.81, p < 0.0001), HOS-Sport (r = 0.55-0.74, p < 0.0001), and NAHS (r = 0.61-0.78, p < 0.0001) measurement tools. PROMIS Computer Adaptive Testing PI T-Score and VAS also demonstrated a strong correlation (r = 0.64-0.80, p < 0.0001).

Conclusions

PROMIS PF scores correlate strongly with mHHS, HOS-ADL, HOS-Sport, and NAHS scores at all time points. Likewise, PROMIS PI scores correlate strongly with VAS pain scores. On average, patients completing PROMIS need to fill out only four or five questions. This study supports the use of PROMIS as an efficient, valid outcome tool for patients with FAI undergoing hip arthroscopy.

## Introduction

The definition of value in the United States healthcare system has recently undergone a paradigm shift with increasing focus placed on patient-reported outcomes (PROs) [1,2]. PROs offer a quantitative analysis of patients’ perceptions of their own health, function, and quality of life. Clinicians, researchers, administrators, and policy-makers are interested in PROs as an aid in determining which interventions provide value. However, many PROs are inconsistently utilized across healthcare systems as various types of measures may have variable applicability to certain conditions. For example, a patient undergoing a total hip arthroplasty in their later years will likely have lower functional demands than a young athlete undergoing hip arthroscopy for femoral acetabular impingement (FAI). As a result, some measures may be susceptible to floor and ceiling effects when applied to different conditions [3-8].

In an effort to improve and standardize PROs, the National Institute of Health developed the Patient-Reported Outcome Measurement Information System (PROMIS). To improve the ease, efficiency, and accuracy of data collection, Computer Adaptive Testing (CAT) was developed as a delivery system for PROMIS [9]. The CAT testing method addresses the inherent limitations of PROs collection through traditional long and short forms. Compared to other collection methods, CAT has demonstrated reduced completion times and has shown to limit floor and ceiling effects [8,10].

PROMIS CAT is used routinely in many healthcare systems, and its applicability has been validated for many disease processes and clinical scenarios. To validate PROMIS in hip arthroscopy, it must be correlated with known legacy measures. The Hip Outcome Score-Activities of Daily Living and Sport subscales (HOS-ADL and HOS-Sport), Nonarthritic Hip Score (NAHS), modified Harris Hip Score (mHHS), and Visual Analog Scale (VAS) have served as traditional legacy measures in this patient population. There have been other studies of small sample sizes that compared PROMIS scores in FAI syndrome to mHHS, international hip outcome tool-33, and the Hip Disability and Osteoarthritis Outcome Score [11-14]. The purpose of this study was to validate PROMIS against traditional legacy measures in patients undergoing hip arthroscopy for FAI. Further, we aimed to establish if floor or ceiling effects exist for PROMIS, determine the average number of questions required to complete PROMIS, and establish minimal clinically important difference (MCID) values in this setting.

## Materials and methods

Institutional review board approval was obtained for this study (STU00205020). A power analysis was performed and an estimated sample size of 62 patients was determined to be necessary to demonstrate a significant correlation (r > 0.4) with an alpha level of 0.05 at 90% power. Ultimately, we elected to enroll 100 patients to account for potential loss to follow-up throughout the study. All English-speaking patients aged 18-60 presenting for hip arthroscopy for a diagnosis of FAI were approached for inclusion in the study. Exclusion criteria included patients unable to consent, minors, and those undergoing revision surgery. Age, sex, date of surgery, type of surgery, and diagnosis associated with the surgical encounter were recorded. The enrollment period occurred from April 2017 to July 2018.

Legacy measures for hip arthroscopy patients included the mHHS, HOS-ADL, HOS-Sport, NAHS, and VAS pain scores. These measures, in addition to PROMIS-CAT Physical Function (PF) and Pain Interference (PI) domains, were collected electronically through the secure Research Electronic Data Capture portal prior to surgery and at two, six, and 12 weeks and six months post-operatively [15]. PROMIS and legacy scoring questionnaires have been described previously in patients undergoing operations in the hip, with higher PF scores indicating better function and lower PI scores indicating less pain [16].

Continuous variables were reported as mean ± standard deviation (SD) and compared using Student’s t-test. Correlations between PROMIS and legacy measures were reported as Spearman rank correlation coefficients. Floor and ceiling effects for each score were considered present if >15% of the respondents achieved either the lowest or highest score, respectively. MCID was calculated as half of the SD of the sample, as previously described [17]. Alpha level was set at p < 0.05. All data and statistical analyses were performed using JMP Pro (version 13.0, SAS, Cary, NC, USA).

## Results

A total of 100 patients were enrolled in this study, of which 99 completed all pre-operative questionnaires. The average age of the patients at the time of enrollment was 39.7 ± 12.9 years (range: 18-67 years). The PROMIS PF and PI assessments were completed with a mean of 4.3 ± 1.1 and 4.6 ± 1.9 questions, respectively. This is in contrast to the mHHS, NAHS, and HOS measures which ask 8, 20 and 26 questions, respectively. Baseline pre-operative PROs are listed in Table 1. The pre-operative PROMIS PF mean T-score was 40.9 ± 6.3 and the PROMIS PI mean T-score was 60.7 ± 5.9. Legacy measure mean scores were as follows: mHHS (64.3 ± 16.3), HOS-ADL (60.5 ± 21.5), HOS-Sport (31.7 ± 23.4), NAHS (58.9 ± 19.3), and VAS (5.6 ± 1.9). The mean improvement in each measure at the six-month time point from baseline was significant (p < 0.0001) and as follows: PROMIS PF (9.3 ± 1), PROMIS PI (-8.9 ± 1), mHHS (21.5 ± 2.4), HOS-ADL (22.2 ± 2.7), HOS-Sport (27.3 ± 3.6), NAHS (22.9 ± 2.6), and VAS (-3.2 ± 0.2) (Table 2). MCID thresholds were calculated for PROMIS PF and PI at 4.4 and (-4.25) points, respectively.

**Table 1:** Changes in PRO scores after hip arthroscopy. Patient reported outcome values at baseline and post-operative follow-up. Scores reported as mean ± SD. Change reported as mean ± SE. PROMIS PF, Patient-Reported Outcomes Measurement Information System Physical Function; PROMIS PI, Patient-Reported Outcomes Measurement Information System Pain Interference; mHHS, modified Harris Hip Score; HOS-ADL, Hip Outcome Score-Activities of Daily Living; HOS-Sport, Hip Outcome Score-Sport; NAHS, Nonarthritic Hip Score; VAS, Visual Analog Scale; PRO, patient-reported outcome; SD, standard deviation; SE, standard error

PROMIS PF	Score (mean ± SD)	Change (mean ± SE)	P-Value
Pre-operative (n = 99)	40.9 ± 6.3	-	-
2 weeks (n = 80)	37.4 ± 7.7	-3.6 ± 0.8	<0.0001
6 weeks (n = 85)	41.5 ± 6.4	0.7 ± 0.8	0.39
12 weeks (n = 79)	46.5 ± 6.7	5.9 ± 0.9	<0.0001
6 months (n = 74)	50.4 ± 9.1	9.3 ± 1	<0.0001
PROMIS PI			
Pre-operative (n = 99)	60.7 ± 5.9		-
2 weeks (n = 80)	59.1 ± 8	-1.8 ± 0.9	0.055
6 weeks (n = 85)	56.4 ± 6.7	-4.4 ± 0.9	<0.0001
12 weeks (n = 79)	54.3 ± 6	-6.8 ± 0.8	<0.0001
6 months (n = 74)	51.4 ± 8	-8.9 ± 1	<0.0001
mHHS			
Pre-operative (n = 99)	64.3 ± 16.3	-	-
2 weeks (n = 80)	66.1 ± 17.9	3.1 ± 2.1	0.14
6 weeks (n = 85)	75.4 ± 16	12.2 ± 2	<0.0001
12 weeks (n = 79)	82.2 ± 13.6	18.5 ± 2	<0.0001
6 months (n = 74)	87 ± 12.4	21.5 ± 2.4	<0.0001
HOS-ADL			
Pre-operative (n = 99)	60.5 ± 21.5	-	-
2 weeks (n = 80)	52.3 ± 21	-7.5 ± 2.6	00.0056
6 weeks (n = 85)	66.2 ± 17.7	6 ± 2.4	0.016
12 weeks (n = 79)	81.9 ± 13.1	21.8 ± 2.3	<0.0001
6 months (n = 74)	83.9 ± 22.4	22.2 ± 2.7	<0.0001
HOS-Sport			
Pre-operative (n = 99)	31.7 ± 23.4	-	-
2 weeks (n = 80)	14.8 ± 20.7	-16.8 ± 2.4	<0.0001
6 weeks (n = 85)	23.3 ± 20.5	-8.3 ± 3.6	0.023
12 weeks (n = 79)	45.4 ± 27	14 ± 3.8	0.0004
6 months (n = 74)	59.9 ± 31.7	27.3 ± 3.6	<0.0001
NAHS			
Pre-operative (n = 99)	58.9 ± 19.3	-	-
2 weeks (n = 80)	58.7 ± 18.5	1.3 ± 2.1	0.54
6 weeks (n = 85)	68.7 ± 15.2	10.2 ± 2.4	<0.0001
12 weeks (n = 79)	80.1 ± 13	21.3 ± 2.2	<0.0001
6 months (n = 74)	82.1 ± 20.5	22.9 ± 2.6	<0.0001
VAS			
Pre-operative (n = 99)	5.6 ± 1.9	-	-
2 weeks (n = 80)	3.7 ± 2	-1.9 ± 0.2	<0.0001
6 weeks (n = 85)	3.3 ± 1.7	-2.3 ± 0.2	<0.0001
12 weeks (n = 79)	2.7 ± 1.7	-3 ± 0.3	<0.0001
6 months (n = 74)	2.3 ± 2	-3.2 ± 0.2	<0.0001

**Table 2:** Baseline PRO values reported as mean ± SD. PROMIS PF, Patient-Reported Outcomes Measurement Information System Physical Function; PROMIS PI, Patient-Reported Outcomes Measurement Information System Pain Interference; mHHS, modified Harris Hip Score; HOS-Sport, Hip Outcome Score-Sport; NAHS, Nonarthritic Hip Score; VAS, Visual Analog Scale; PRO, patient-reported outcome; SD, standard deviation

Characteristic	Value
No. of patients	99
Age	39.7 ± 12.9
Pre-operative PROs (mean ± SD)	Mean ± SD
PROMIS PF	40.9 ± 6.3
PROMIS PI	60.7 ± 5.9
mHHS	64.3 ± 16.3
HOS	60.5 ± 21.5
HOS-Sport	31.7 ± 23.4
NAHS	58.9 ± 19.3
VAS	5.6 ± 1.9

Spearman rank correlation coefficients were calculated between PROMIS PF and known legacy measures. Strong correlations were observed between the PROMIS PF T-Score and the mHHS (r = 0.64-0.83, p < 0.0001), HOS-ADL (r = 0.54-0.81, p < 0.0001), HOS-Sport (r = 0.55-0.74, p < 0.0001), and NAHS (r = 0.61-0.78, p < 0.0001) measurement tools at all time points (Table 3). PROMIS PI T-Score and VAS also demonstrated a strong correlation (r = 0.64-0.80, p < 0.0001) at all time points (Table 4).

**Table 3:** PROMIS PF correlation with legacy measures PRO, patient-reported outcome; mHHS, modified Harris Hip Score; HOS-ADL, Hip Outcome Score-Activities of Daily Living; HOS-Sport, Hip Outcome Score-Sport; NAHS, Nonarthritic Hip Score; VAS, Visual Analog Scale; PROMIS PF, Patient-Reported Outcomes Measurement Information System Physical Function

PRO	r	P-Value
mHHS		
Pre-operative	0.83	<0.0001
2 weeks	0.725	<0.0001
6 weeks	0.647	<0.0001
12 weeks	0.649	<0.0001
6 months	0.671	<0.0001
HOS-ADL		
Pre-operative	0.817	<0.0001
2 weeks	0.729	<0.0001
6 weeks	0.71	<0.0001
12 weeks	0.724	<0.0001
6 months	0.544	<0.0001
HOS-Sport		
Pre-operative	0.741	<0.0001
2 weeks	0.559	<0.0001
6 weeks	0.656	<0.0001
12 weeks	0.715	<0.0001
6 months	0.726	<0.0001
NAHS		
Pre-operative	0.789	<0.0001
2 weeks	0.663	<0.0001
6 weeks	0.632	<0.0001
12 weeks	0.744	<0.0001
6 months	0.613	<0.0001

**Table 4:** PROMIS PI correlation with VAS. PRO, patient-reported outcome; VAS, Visual Analog Scale; PROMIS PI, Patient-Reported Outcomes Measurement Information System Pain Interference

PRO	r	P-Value
VAS		
Pre-operative	0.731	<0.0001
2 weeks	0.737	<0.0001
6 weeks	0.646	<0.0001
12 weeks	0.706	<0.0001
6 months	0.802	<0.0001

Floor and ceiling effects were calculated for each PROMIS and legacy measure, and no significant effects were observed. Each instrument had the following floor and ceiling effects: PROMIS PF 0% and 1.7%, PROMIS PI 7.1% and 0%, mHHS 0% and 5.4%, HOS-ADL 0% and 4.8%, HOS-Sport 10.7% and 3.2%, NAHS 0% and 2.4%, and VAS 7.4% and 0.4%, respectively.

## Discussion

This study indicates PROMIS PI and PF scales demonstrate convergent validity and responsiveness with legacy measures for patients undergoing hip arthroscopy for FAI. Further, these scales distinguish clinical improvements in an efficient manner without notable floor or ceiling effects.

PROMIS CAT utilizes item response theory to minimize redundancy and maximize precision in PRO measurement collection by customizing future questions based on prior responses [18]. This eliminates the need for patients to complete all the questions to attain a valid test score unlike standard legacy measures. In this series, the PROMIS scores were obtained with less than five questions on average. This is compared to the mHHS, NAHS, and HOS measures which ask eight, 20, and 26 questions, respectively. Longer questionnaire length has been linked to decreased rate and quality of responses, though to our knowledge, has not been assessed for traditional hip legacy measures [19]. Although our study did not directly assess time to completion, other PROMIS studies have indicated reduced number of questions correlated with shorter time to completion [20,21].

Previously described hip legacy measures have demonstrated floor and ceiling effects in hip preservation surgery. Kemp et al. reported notable ceiling effects for both mHHS (24%) and HOS-ADL (16%) between 12 and 24 months after surgery [22]. PROMIS PI and PF were able to accurately assess physical function and pain status without notable floor or ceiling effects. This is consistent with other reports in the literature of pre-operative PROMIS values for FAI patients undergoing hip arthroscopy [14]. This indicates that the PROMIS item response question selection accurately and precisely quantifies patient pain and function in the FAI cohort. The efficiency of the PROMIS CAT system allows for data collection in a productive manner prior to a clinical encounter either at home or in a waiting room with immediate scoring that is available during a clinical encounter. Further, these scores can be utilized by physicians or other providers to quantify baseline functional or pain levels and change overtime in either operative or non-operative interventions. Additionally, patients can use this information to track their own personal health progress.

This study also sought to quantify MCID values using a distribution-based method, as described in previous orthopedic surgery studies [23,24] PROMIS MCID PF and PI values were 4.4 and -4.25 points, respectively. The mean improvement in each measure at the six-month time point from baseline was statistically significant (p < 0.0001) as well as clinically significant following the above MCID thresholds at PROMIS PF (9.3 ± 1), PROMIS PI (-8.9 ± 1).

Despite the benefits of PROMIS testing, our study also has several limitations. Even though adequate power for clinical correlation was obtained, our study is limited in sample size for more thorough subgroup analysis. As such, much of the baseline demographics and specifics of the surgical procedure were not included in this study. Further, while we are still collecting additional data, one and two years post-surgical intervention, we felt that the five data points would be more than sufficient for validation of the metric as well as determining the average number of questions needed to complete PROMIS metrics. We also report MCID values using a distribution-based method which has been criticized for its generalizability, though to date, there is no generally accepted method for calculating MCID. Lastly, our study included only adults and English-speaking patients and may not be generalizable to non-English speakers or patients under the age of 18.

## Conclusions

PROMIS PF scores correlate strongly with mHHS, HOS-ADL, HOS-Sport, and NAHS scores at all time points. Likewise, PROMIS PI scores correlate strongly with VAS pain scores. On average, patients completing PROMIS need to fill out only four or five questions, which can help reduce survey fatigue and possibly improve quality. This study supports the use of PROMIS scores as an efficient, valid physical function and pain assessment tool for patients with FAI undergoing hip arthroscopy.
